# Impact of Illness Perception in Overweight and Obesity on Bio-Functional Age and Eating/Movement Behavior—A Follow-Up Study

**DOI:** 10.1089/whr.2024.0012

**Published:** 2024-10-10

**Authors:** Tiziana Felicitas Aimée Marti, Elena Pavicic, Linda Maria Roggo, Norman Bitterlich, Michael von Wolff, Dagmar Poethig, Petra Stute

**Affiliations:** ^1^ University of Bern, Bern, Switzerland.; ^2^ Draisdorfer Straße 21, Chemnitz, Germany.; ^3^ Department of Gynaecologic Endocrinology and Reproductive Medicine, University Clinic of Obstetrics and Gynaecology, Inselspital Bern, Bern, Switzerland.; ^4^ European Association on Vitality and Active Aging eVAA e.V., EIP-AHA Reference Site Saxony, Leipzig, Germany.

**Keywords:** illness perception, overweight, obesity, bio-functional age, aging, sense of coherence

## Abstract

**Background:**

Despite the widespread prevalence of obesity and its potential adverse impacts on health, the majority of interventions aimed at weight loss stay ineffective. This study aimed to assess illness perception in people with overweight/obesity and its impact on bio-functional age (BFA) and cognitive patterns governing eating and movement behavior.

**Methods:**

A total of 40 subjects from the original overweight/obesity subcohort of the Bern Cohort Study 2014 (BeCS14) were included and assessed for a follow-up from 2019-11-29 to 2020-07-14. The subjects completed a validated “bio-functional status” test battery with calculation of BFA, as well as validated questionnaires for eating and movement behavior and illness perception.

**Results:**

Participants were overall bio-functionally younger than their chronological age (mean 4.3 ± 6.9 year equivalents) but aging was more pronounced than anticipated. Mental occupation with illness cause was moderate to high with psychosocial (PS) factors being more pronounced than naturalistic (NT) factors. There was a shift from defined theory with focus clearly on PS theories to diffuse theory with consideration of both PS and NT theories. Participants with good sense of coherence (SOC) were less likely to be mentally preoccupied with illness cause (*p* < 0.05, *r_s_* = −0.404), especially with PS factors. PS theories on illness cause correlated with pathological eating behavior (emotional eating: *p* > 0.05, *r_s_* = 0.378; temptation: *p* < 0.01, *r_s_* = 0.486).

**Conclusions:**

Illness perception does affect cognitive patterns and integrating it into therapeutic management for people with obesity can enhance outcomes. Strengthening of SOC is important to decrease PS stress and achieve better subjective health, less mental preoccupation, and less dysfunctional eating behavior.

## Introduction 

Overweight and obesity have become a major global health concern, even though obesity is preventable. High body mass index (BMI) is a risk factor for many noncommunicable diseases (NCD) such as diabetes, cardiovascular diseases, and some cancers.^1^ Mortality increases in people with BMI above a certain threshold, and there is evidence that body fat percentage correlates with bio-functional pro-aging.^2,3^ Therefore weight loss appears to be a sensible approach to decrease health risks in people with overweight or obesity.^1^ Nevertheless, the realization of weight loss in the context of prevention and therapy still poses difficulties.

When faced with a health threat, people develop cognitive models of this threat which determine their response. For other medical conditions such as cardiovascular diseases, patients’ perception of the disease has been found to have a several impacts on therapeutic outcome.^4^ Although cognitive patterns (*e.g.,* emotional eating, craving, disinhibition) are known to regulate eating and movement behavior, perceived illness causes for overweight and obesity have barely been assessed before.^5–6^ Studies have shown that gender and subjective illness representation play an important role for weight-related variables, while females have more unfavorable values for the latter.^7^ Women exhibited a greater prevalence of maladaptive illness representations than men, and in the context of obesity, illness representations are influenced by experiences related to weight cycling.^8^

In the Bern Cohort Study 2014 (BeCS-14), eating and movement behavior and illness perception of obesity as well as bio-functional age (BFA) in nonpediatric, nongeriatric females was assessed.^9^ Higher chronic stress exposure was associated with bio-functional pro-aging and less vitality.^10^

Based on the results of the first measurements in 2014, confirmation that participants with psychosocial (PS) stress exhibit pathological eating and movement behavior was expected. Another hypothesis was that participants with negative illness perceptions and high mental occupation experience pro-aging. In order to evaluate these hypotheses, the objective of this study was to (1) assess illness perceptions in the BeCS-14 subcohort of people with overweight or obesity, (2) analyze if illness perception in this subcohort is associated with BFA, and (3) examine correlations between illness perception and cognitive patterns that affect regulation of eating and movement behavior.

## Subjects, Material, and Methods

### Study design

The present analysis was a follow-up of the BeCS-14 and included calculation of participants’ BFA, as well as exploration of their eating and movement behavior and illness perception of obesity. The tools applied were based on a complex Active and Healthy Aging (AHA) assessment model while incorporating the International Classification of Functioning, Disability and Health (ICF) concept and classification.^9,11,12^ After the first examination in 2014, participants were sensitized to the subject of AHA and got to know their strengths and resources in an individual discussion of their results; however, there was no intervention.

BeCS-14 is a single-center, cross-sectional, observational, noninterventional, and nonrandomized trial in German-speaking women of age 18–65 years. Exclusion criteria were acute diseases, pregnancy, and illiteracy. At first, all participants completed the same standardized base test battery, which comprised personal and family history, bio-functional status (BFS), and BFA, as well as validated questionnaires for depression and anxiety (Hamilton Depression Scale), health-related quality of life (Short Form-36 Health Survey), and chronic stress (*Trierer Inventar zur Erfassung von chronischem Streß*).^13–16^ Subsequently, participants were asked to take part in one of four nonrandomized subgroups, of which one subgroup (nutrition) comprised participants with overweight or obesity only (BMI ≥25 kg/m^2^). In this subgroup, participants completed two additional validated questionnaires addressing eating and movement behavior (*Interdisziplinäres Testsystem zur Diagnostik und Evaluation bei Adipositas und anderen durch Ess-und Bewegungsverhalten beeinflussbaren Krankheiten*, Assessment of Eating and Movement Behavior [AD-EVA]) and illness perception (Patiententheoriefragebogen, PATEF).^5,17^

Recruitment of the original participants of subgroup “nutrition” for a follow-up commenced on 2019-11-27. For all participants at least 5 years had passed since baseline measurements. Surveys and examinations were performed from 2019-11-29 to 2020-07-14 at the Department of Obstetrics and Gynaecology, Inselspital University Bern, Switzerland.

Of 99 participants, 40 participants (40.4%) were successfully recruited for follow-up assessment. The remaining participants either passed away in the meantime (*n* = 2), did not want to be contacted for a follow-up (*n* = 4), declined the invitation to participate (*n* = 20), or did not respond (*n* = 33).

### Assessment procedures

#### Personal and family history

Participants’ information, lifestyle habits, and personal history, as well as family history, were surveyed in brief. For this follow-up, questions about changes since the last assessment were added to the questionnaire.

#### Bio-functional status and bio-functional age^13^

The BFS is a generic age- and sex-specific test battery taking into account physical, mental-emotional, and social characteristics. By incorporating the ICF and AHA concepts, respectively, it allows the identification of an individual’s strengths and health resources (Supplementary Data S1).^13^

The summation of BFS single values makes up the BFA Index (BFAI). An individual’s BFAI can be transformed into BFA using gerontologic aging tables, which were developed from the reference population. BFA is measured in years and can be compared to the chronological age (CA). BFA < CA indicates above-average body function for a person’s age, whereas BFA > CA suggests pro-aging.

#### Patiententheoriefragebogen (patient theory questionnaire)^17^

PATEF is a validated questionnaire addressing patient’s illness perception. It examines to what extent perceived illness cause is naturalistic (NT), PS, or attributed to health behavior (HB). Overall, it comprises 46 items covering five subscales. Each item is rated on a five-point rating scale. Based on norm charts, raw values are converted into age- and sex-validated *S*-values (range 1–9) for all subscales.^17^

PS subscales: PS external (PSE) covers stress due to social environment factors, such as family problems, working atmosphere or conflict with other people. *S* > 6 indicates high risk for helplessness and depression. PS internal (PSI) covers strain due to inner discontent and low self-esteem. *S* > 6 corresponds to patients’ belief to be responsible for their state of health due to inability to take control over their own life.

NT subscales: NT external covers mostly uncontrollable, harmful environmental factors such as climate change and air pollution. *S* > 6 may indicate either fatalistic attitude and limited cooperation with treating physician or, on the contrary, belief of powerlessness with the sole hope of their doctor’s “miracle therapy.” NT internal (NTI) covers the body as primary cause of disease, for example, due to inherited predisposition. *S* > 6 signifies passive attitude and high trust in drug treatment with good compliance.

HB subscale covers behavior with adverse health effects. *S* > 6 indicates feeling responsible for health deterioration due to self-inflicted unhealthy behavior.

Overall score (OS) is an index for general intensity of mental occupation with illness cause. *S* > 6 corresponds to high mental occupation with illness cause, whereas *S* < 4 corresponds to no excessive thoughts or theory not covered by PATEF. Subjects may have an already established theory on illness cause (1–3 subscales with *S* > 6) or diffuse theories (4–5 subscales with *S* ≥ 5).

#### Interdisziplinäres testsystem zur diagnostik und evaluation bei adipositas und anderen durch ess-und bewegungsverhalten beeinflussbaren krankheiten (AD-EVA)^5^

AD-EVA is a validated questionnaire addressing eating and movement behavior. It comprises 153 items divided into nine scales. Items are rated on a point rating scale or based on a yes/no rating scale. Raw values are converted into comparable *T*-values (range 20–80) based on norm charts differentiating between BMI groups. *T*-values 40 to <60 are considered average, whereas *T*-values <30 or >70 are considered extreme.**^5^**

The scale Salutogenic eating behavior (FEV-Salute) covers health-promoting behavior, such as ability to control eating schedule and amount of food eaten (FEV-Salute-MK), regular physical activity (FEV-Salute-S), compliance with commonly known recommendations concerning food and exercise (FEV-Salute-EU), and relaxed handling of food with ability to enjoy (FEV-Salute-GE).

The scale Pathogenic eating behavior (FEV-Path) comprises unhealthy behavior, such as deliberate caloric restriction and avoiding of certain foods in order to lose weight (FEV-Path-K), influence of temptations on behavior (FEV-Path-S), and desire to eat whenever negative emotions occur (FEV-Path-EE).

The scale Handling of food (FUN) covers excessive eating by assessing craving and dependence. Both clinical eating disorder (FBEB), such as binge eating disorder and bulimia, and preclinical eating disorder (FVE-PWS), indicated by preoccupation with weight and shape, are covered in this questionnaire. There are also subscales concerning Quality of life (SLQ), movement motivation (FBM), dietary preferences (EPL) of healthy food, and hearty food and snacks, as well as body image (SKB), that assess self-perception and aspired ideal.

### Statistics

Statistical analysis was performed with Statistical Package for the Social Sciences version 27. The descriptive statistics included the calculation of the mean, standard deviation, and range (minimum, maximum), as well as the 5th and 95th percentiles. The Wilcoxon signed-rank test was used to assess the results of the first and second measurement for significant differences, and effect size was calculated for scales with statistically significant difference (*p* < 0.05). Correlation statistics were performed by means of Spearman’s rank correlation coefficient with a significance of 5% (two-sided) or 2.5% (one-sided). No sample size calculation was performed, since for this noninvasive study, the aim was to achieve the highest response rate possible not to include a certain number of participants.

## Results

### Characteristics of the cohort

In total, 40 subjects (85% female, 15% male) of the “nutrition” subgroup of the BeCS-14 cohort were seen a second time and considered for this study. Mean age was 54.4 ± 11.4 years and mean BMI was 30.4 ± 5.0 kg/m^2^. The majority of recruited subjects were people with overweight with BMI 25–29.9 kg/m^2^ (42.5%) or were people with obesity with BMI ≥ 30 kg/m^2^ (47.5%). Even though more participants had reached a normal BMI compared to the first measurement, there were now more extreme BMI values in both directions. The characteristics of the cohort are presented in Table 1. The number of cases is limited by the recruitment possibilities of the repeat measurement (after 6 years). With 40 subjects, a Cohen’s effect size of approximately 0.50 (medium effect) can be achieved with the Wilcoxon test for linked samples for 5% significance level and 80% power. The medical history questionnaires were different in 2014 and 2020, so not all questions were submitted for 2014. The difference of nine in the number of participants can be explained by the fact that some questions were not asked in the 2014 medical history. However, as these are not included in the statistics, no distorting influences are to be expected.

**Table 1.** Characteristics of the Cohort—Comparison Between Second (M02) and First (M01) Measurement

Parameter	M02 (*n* = 40)	M01 (*n* = 31)
	%	%
Sex		
Female	85	85
Male	15	15
Weight		
Underweight (BMI < 18.5 kg/m^2^)	2.5	0
Normal weight (BMI 18.5–24.9 kg/m^2^)	7.5	2.5
Overweight (BMI 25–29.9 kg/m^2^)	42.5	50
Obesity I (BMI 30–34.9 kg/m^2^)	25	35
Obesity II (BMI 35–39.9 kg/m^2^)	20	10
Obesity III (BMI ≥ 40 kg/m^2^)	2.5	2.5
Graduation		
High school/middle school	0	9.7
Apprenticeship/professional school/commercial school	42.5	19.4
Diploma school/teacher’s seminar	10	9.7
Federal certificate/professional diploma/examination for master craftsman’s certificate/higher commercial school	17.5	32.3
Higher education (*e.g.,* university)	30	29
Employment status		
Full time (≥90%)	35	48.4
Part time (50%–89%)	35	29
Part time (<50%)	7.5	16.1
Retired	22.5	0
Jobless (including students)	0	6.5
Income (gross income, per month)		
<5,000 cHF	42.5	48.4
5,000–10,000 cHF	47.5	41.9
>10,000 cHF	10	9.7
Working despite feeling sick (last 12 months)		
Never	17.5	45.2
Once	20	16.1
Several times	37.5	38.7
Answer missing	25	0
Sick days (last month)		
0 days	50	71
1 or more days	25	29.1
Answer missing	25	0
Stress due to sick days		
Yes	22.5	12.9
No	50	87.1
Answer missing	27.5	0
Satisfaction with job situation		
Very low	0	—
Low	0	—
Medium	22.5	—
High	42.5	—
Very high	27.5	—
Change of job position since 2014		
No	62.5	—
Yes	37.5	—
Current living situation		
Single, living alone	20	19.4
Single, not living alone	7.5	12.9
Partnership/married	67.5	58.1
Divorced/widowed	5	9.7
Number of children		
None	30	29
1 child	12.5	16.1
2 or more children	57.5	54.9
Satisfaction with partnership		
Very low	2.5	0
Low	10	3.2
Medium	7.5	12.9
High	17.5	32.3
Very high	62.5	51.6
Satisfaction with sexuality		
Very low	10	9.7
Low	5	6.5
Medium	10	25.8
High	40	29
Very high	35	29
Type of nutrition		
Balanced	87.5	—
Vegan	2.5	—
Others	10	—
Change of nutrition type since 2014		
Yes	25	—
No	75	—
Alcohol frequency		
Never	10	9.7
Once a month	22.5	9.7
2–4 times per month	30	38.7
2–3 times per week	32.5	38.7
4 or more times per week	5	2.5
Alcohol amount a day (number of standard glasses)		
0	67.5	64.5
1	15	12.9
2 or more	17.5	22.5
Smoking		
Never smoked	55	51.6
Ex-smoker	32.5	38.7
At least 10 cigarettes per day	12.5	9.7
Sports		
Rarely	15	9.7
<1 time per week	17.5	25.8
1–2 times per week	37.5	51.6
>2 times per week	30	12.9
Change of sport habits since 2014		
More sports	32.5	—
Same amount	35	—
Less sports	32.5	—
Sleep duration (average, last 4 weeks)		
≤5 hours	5	0
6 hours	25	32.3
7 hours	57.5	48.4
≥8 hours	12.5	19.4
Sleep quality		
Very good	22.5	19.4
Rather good	57.5	64.5
Rather bad	20	12.9
Very bad	0	3.2
Snoring		
Yes	45	51.6
No	30	35.5
Do not know	25	12.9
Change in health situation (last 2 years)		
Yes	40	—
No	60	—
Loss of mobility (last 2 years)		
Yes	17.5	—
No	82.5	—
Change of family health situation (last 2 years)		
Yes	32.5	—
No	67.5	—
Number of hospitalizations (last 12 months)		
0	85	—
1	2.5	—
>1	12.5	—
Personal and family history		
Currently disease free	10	—
Cardiovascular family history	62.5	—
History of cancer	22.5	—
Number of medications		
None	27.5	—
1	20	—
2	25	—
3	10	—
>3	17.5	—
Most common medications		
Vitamins and supplements	30	—
Antihypertensive medication	25	—
Phytopharmaceuticals	15	—
Psychotropic medication	12.5	—
Sexual steroid hormones	5	—
Analgetic medication	5	—

BMI, body mass index.

### Bio-functional status and bio-functional age

In accordance with participants growing older (mean +5.8 years) since the first measurement, BFA increased as well (mean +9.5 years). Although average BFA was still lower than CA, the difference between the two decreased from 8.7 ± 8.5 years to 4.3 ± 6.9 years. Since a small difference between CA and BFA corresponds to a less functional, “older” body, participants seem to have aged more than was expected (Supplementary Data S2).

Most physical parameters, sensory physiology, and psychomotor parameters, as well as cognitive and mental abilities deteriorated. On the other hand, the majority of emotional–social parameters did not notably change since the first measurement.

### Eating and movement behavior (AD-EVA)

BMI varied widely, ranging from underweight (BMI < 18.5 kg/m^2^) to obesity III (BMI ≥ 40 kg/m^2^); 90% of participants were people with either overweight or obesity. About 80% of participants had tried at least one measure against overweight in their life, and most had even tried several. Diet was most frequently mentioned (55%) followed by nutrition counseling (42.5%) (Supplementary Data S2).

Overall, the majority of AD-EVA questionnaire parameters revealed average *T*-values. However, below-average *T*-values in the subscale FBM-S&B indicated that movement motivation through fun and satisfaction was lower than that in a representative sample. Furthermore, above-average *T*-values in the subscale EPL-G revealed a fondness for healthy foods. Compared to the first measurement, participants got significantly better at restraining themselves when faced with temptations, which is reflected in a decrease in FEV-Path-S *T*-value.

### Illness perception (patiententheoriefragebogen)

*S*-value of OS was either moderate (58.9%) or high (33.4%) in most participants, indicating high mental occupation with illness cause. Mean *S*-value was highest in the subscale HB (6.41 ± 1.73) and higher in PS subscales (6.18 ± 1.41) than in NT subscales (5.54 ± 1.67) (Table 2; Supplementary Data S2).

**Table 2.** Prevalence of Mental Occupation with Overweight/Obesity and Focus of Illness Cause (PATEF)-Comparison Between Second (M02) and First (M01) Measurement

Parameter	M02	M01	Difference mean value M02–M01	Difference statistically relevant (*p* < 0.05)
	Mean (SD)	Mean (SD)		
Psychosocial external (PSE)	6.15 (1.18)	5.88 (1.20)	0.27	
Psychosocial internal (PSI)	6.08 (1.33)	6.25 (1.41)	−0.17	
Psychosocial (PS)	6.18 (1.41)	5.98 (1.39)	0.20	
Naturalistic external (NTE)	5.18 (1.52)	4.40 (1.17)	0.78	*Z* = −3.154, *p* = 0.002, *r* = 0.3571
Naturalistic internal (NTI)	5.59 (1.45)	5.28 (1.38)	0.31	
Naturalistic (NT)	5.54 (1.67)	4.75 (1.48)	0.79	*Z* = −2.973, *p* = 0.003, *r* = 0.3366
Health behavior (HB)	6.41 (1.73)	6.53 (1.71)	−0.12	
Overall score (OS)	5.69 (1.66)	5.48 (1.47)	0.21	
		
Number of *S*-values >5	4.87 (2.80)	4.13 (2.97)	0.74	*Z* = −2.276, *p* = 0.023, *r* = 0.2577
Number of *S*-values >6	2.87 (2.98)	2.28 (2.21)	0.59	

Subscales PS internal and HB both improved, meaning that participants were more content and attributed less health problems to self-inflicted behavior. However, all other parameters deteriorated from first to second measurement. The increase of natural external factors as perceived illness cause was highly significant, with mean *S*-value increasing from 4.40 ± 1.17 at first to 5.18 ± 1.52 at second measurement (*p* = 0.002). This indicates that participants found uncontrollable, harmful environmental factors much more likely to be responsible for health deterioration. There was a shift from participants having a clear focus on PS scales to them considering both PS and NT theories to be very likely.

#### Subgroup analysis for data grouped by age, grade of aging, and BMI

Participants under the age of 45 years achieved a higher OS (6.7 ± 1.9) than older participants (5.5 ± 1.6), which suggests increased mental occupation with possible illness cause in younger participants. This outcome was already shown at the first measurement but became even more pronounced (Table 3).

**Table 3.** PATEF Group Analysis-Comparison Between Second Measurement (M02, Upper Value) and First Measurement (M01, Lower Value)

	*T*-value of PATEF subscales: Mean (SD)							
	PSE	PSI	HB	NTE	NTI	PS	NT	OS
Grouped by age								
<45 years (*n* = 7)	6.7 (1.0) 6.1 (0.7)	6.4 (1.5) 6.7 (1.3)	7.4 (1.4) 7.6 (1.4)	5.7 (1.8) 5.4 (0.8)	6.6 (1.4) 5.7 (1.0)	6.7 (1.1) 6.4 (1.0)	6.6 (1.9) 5.6 (1.1)	6.7 (1.9) 6.3 (1.3)
≥45 years (*n* = 33)	6.0 (1.2) 5.8 (1.3)	6.0 (1.3) 6.2 (1.4)	6.2 (1.7) 6.3 (1.7)	5.1 (1.5) 4.2 (1.1)	5.4 (1.4) 5.2 (1.5)	6.1 (1.5) 5.9 (1.5)	5.3 (1.6) 4.6 (1.5)	5.5 (1.6) 5.3 (1.5)
Grouped by grade of aging								
Pro-aging (*n* = 4)	5.0 (1.0) 5.3 (1.3)	5.3 (1.5) 5.8 (1.5)	4.3 (0.6) 6.3 (1.7)	4.0 (1.0) 4.0 (0.8)	4.7 (1.5) 5.0 (0.8)	5.0 (2.0) 5.3 (1.7)	4.0 (2.0) 4.7 (0.5)	4.0 (2.0) 5.0 (1.4)
Physiological aging (*n* = 22)	6.1 (1.2) 5.6 (1.3)	5.9 (1.4) 6.0 (1.5)	6.2 (1.8) 6.0 (1.7)	5.3 (1.4) 4.0 (1.1)	5.3 (1.5) 5.0 (1.4)	6.0 (1.5) 5.7 (1.5)	5.5 (1.6) 4.3 (1.5)	5.5 (1.6) 5.1 (1.4)
Slow aging (*n* = 8)	6.3 (1.2) 6.6 (0.7)	6.5 (0.8) 6.9 (1.1)	7.0 (1.2) 7.4 (1.2)	5.0 (1.6) 5.0 (1.2)	5.9 (0.8) 6.0 (1.7)	6.8 (0.9) 6.9 (0.6)	5.6 (1.3) 5.5 (1.5)	6.1 (0.8) 6.3 (1.4)
Grouped by BMI group								
Group 1: BMI <19.0 kg/m^2^ (*n* = 1)	7.0 (0.0) 7.0 (0.0)	9.0 (0.0) 9.0 (0.0)	9.0 (0.0) 9.0 (0.0)	5.0 (0.0) 4.0 (0.0)	8.0 (0.0) 5.0 (0.0)	8.0 (0.0) 8.0 (0.0)	7.0 (0.0) 4.0 (0.0)	9.0 (0.0) 8.0 (0.0)
Group 2: BMI 19.0–24.9 kg/m^2^ (*n* = 3)	4.5 (0.7) 5.0 (1.7)	5.0 (1.4) 5.3 (1.5)	4.5 (0.7) 4.7 (1.2)	4.0 (1.4) 3.7 (1.2)	4.5 (2.1) 4.0 (1.0)	4.5 (2.1) 4.7 (2.1)	3.5 (2.1) 3.7 (1.5)	3.5 (2.1) 4.0 (1.7)
Group 3: BMI 25.0–29.9 kg/m^2^ (*n* = 17)	6.1 (1.1) 5.7 (1.2)	5.8 (1.1) 6.1 (1.0)	6.2 (1.4) 6.1 (1.6)	5.2 (1.6) 4.4 (1.4)	5.4 (1.5) 5.2 (1.4)	6.1 (1.0) 5.9 (1.1)	5.4 (1.9) 4.5 (1.7)	5.5 (1.3) 5.2 (1.2)
Group 4: BMI 30.0–34.9 kg/m^2^ (*n* = 10)	6.1 (1.2) 6.2 (1.2)	6.1 (1.6) 5.9 (1.6)	6.5 (2.2) 7.1 (1.5)	5.4 (1.7) 4.4 (1.1)	5.8 (1.3) 5.3 (1.1)	6.2 (1.8) 6.0 (1.5)	5.8 (1.5) 4.9 (1.1)	5.8 (1.8) 5.6 (1.5)
Group 5: BMI 35.0–39.9 kg/m^2^ (*n* = 8)	6.5 (1.4) 6.0 (1.1)	6.4 (1.2) 7.0 (1.5)	6.9 (1.7) 7.0 (1.8)	4.9 (1.4) 4.6 (0.9)	5.5 (0.9) 5.6 (1.7)	6.5 (1.6) 6.4 (1.5)	5.5 (1.1) 5.3 (1.3)	6.0 (1.6) 5.9 (1.6)
Group 6: BMI ≥40.0 kg/m^2^ (*n* = 1)	7.0 (0.0) 6.0 (0.0)	7.0 (0.0) 7.0 (0.0)	7.0 (0.0) 8.0 (0.0)	7.0 (0.0) 6.0 (0.0)	8.0 (0.0) 7.0 (0.0)	7.0 (0.0) 6.0 (0.0)	8.0 (0.0) 7.0 (0.0)	7.0 (0.0) 7.0 (0.0)

Grade of aging was determined for all participants by comparing the difference between chronological and BFA between first and second measurement. OS was much lower in pro-aging participants (4.0 ± 2.0) than in physiologically aging participants (5.5 ± 1.6) and participants with healthy “slow” aging (6.1 ± 0.8). Therefore, pro-aging was associated with less excessive thinking about possible illness causes. This effect was particularly strong in the subscale HB.

OS, and therefore mental preoccupation, was higher with more extreme BMI values—in both directions, underweight and obesity.

### Impact of illness perception (patiententheoriefragebogen) on eating and movement behavior (AD-EVA)—and vice versa

OS of PATEF and therefore the degree of mental occupation with illness cause was higher in participants with pathogenic eating behavior who struggled with disinhibition, cognitive control, and emotional eating. Accordingly, OS was lower in participants with salutogenic eating behavior, especially in subjects with high mean control. The strongest correlation with OS was found to be FUN (correlation coefficient 0.468, *p* > 0.01), showing that excessive eating correlates with negative theories and higher general mental occupation (Table 4; Supplementary Data S3).

**Table 4.** Ranking of AD-EVA Parameters with High Correlation (*p* < 0.01) to PATEF Subscales

Rank	AD-EVA parameter	PATEF subscale	Correlation to PATEF scale: Spearman correlation coefficient
1	FUN	NTI	0.559
2	FUN	PSI	0.528
3	FEV-Salute-MK	PSI	−0.502
4	FEV-Path-EE	NTI	0.479
5	FEV-Salute-MK	HB	−0.474
6	FEV-Path-S	PSI	0.437
7	FVE	HB	0.434
8	FEV-Path-K	NTI	0.430
9	FEV-Path-S	PSE	0.419
	FEV-Path-S	NTI	0.419
10	FEV-Salute-MK	PSE	−0.412

Both PS external and PS internal subscales, as well as HB, mostly correlated with eating behavior. They demonstrated positive correlations with pathogenic and negative correlations with salutogenic eating behavior. Therefore, healthier eating behavior was exhibited in participants who did not attribute health deterioration to stress due to environmental factors (PSE), inner discontent (PSI), or adverse HB. Correlation of these PATEF subscales was especially strong, with the subscale assessing the ability to control the amount of food eaten (FEV-Salute-MK). All FEV-Path scales, as well as FUN (handling of food), correlated highly positively with subscale NTI, indicating that participants with pathogenic eating behavior found their body to be a likely cause of disease. FUN also showed high correlation with PS internal subscale, which indicates stronger craving for and dependence on excessive eating in participants with low self-esteem and inner dissatisfaction. Participants preoccupated with weight and shape (subscale FVE) seemed to hold themselves responsible for health deterioration due to behavior with adverse health effects (subscale HB).

Correlation with OS was similar in both measurements, and PS scales of PATEF correlated with pathological eating behavior in both measurements as well.

### Impact of illness perception (patiententheoriefragebogen) on bio-functional age—and vice versa

OS of PATEF correlated strongly with physical, emotional, and overall well-being. The more problems in the area of well-being, comprising factors such as stress regulation and social interaction, the higher was the mental occupation with illness cause theories. These correlations with PATEF mainly stemmed from subscales PS internal and HB. Therefore, low well-being correlated with inner discontent, low self-esteem, and feeling responsible for behavior with adverse health effects (Table 5; Supplementary Data S3).

**Table 5.** Ranking of BFA Parameters with High Correlation (*p* < 0.01) to PATEF Subscales

Rank	BFA parameter	PATEF subscale	Correlation to PATEF scale: Spearman correlation coefficient
1	Sense of coherence	PSI	−0.620
2	Test motivation	PSE	0.573
3	Test motivation	HB	0.547
4	Hearing loss left 4096 Hz	HB	−0.519
5	Test motivation	NTI	0.504
6	Emotional well-being	PSI	0.485
7	Test motivation	PSI	0.475
8	Concentration (time)	NTI	0.473
9	Resting heart rate	NTE	0.447
10	Cognitive reaction time	HB	−0.440
11	Psychomotor endurance	HB	0.435
12	Overall well-being	PSI	0.433
13	Emotional well-being	HB	0.424
14	Chronological age	HB	−0.413
	Stress exposition	PSI	−0.413
15	Overall well-being	HB	0.412

BFA, bio-functional age.

Altogether, the most pronounced correlation, measured by the amount of the correlation coefficient, was the negative correlation of PS internal subscale with sense of coherence (SOC) (correlation coefficient −0.620; *p* < 0.01). According to these findings, participants with inner discontent and low self-esteem who found themselves unable to take control of their own life had a lower SOC with less helpful coping strategies and less effective problem-solving skills. Moreover, there was a negative correlation of HB with CA (correlation coefficient −0.413, *p* < 0.01), indicating that older participants were less likely to suspect adverse HB to be a possible cause of illness.

Comparing the first to the second measurement, low emotional well-being and low SOC were linked with high mental occupation with illness cause, especially with PS internal theories.

## Discussion

In accordance with the physiological aging process, a certain age-related deterioration in BFS parameters could be expected. However, as demonstrated by a decrease in the difference between chronological and BFA of the participants, this deterioration was more severe than that attributed to the aging process. This means that the participants of this study showed signs of pro-aging. No factors with particularly strong contribution to pro-aging in the sense of a “risk factor” for premature aging could be determined in this study’s patient population (namely, no stress or illness perception or lifestyle). However, participants with a higher number of diseases were more likely to experience pro-aging. This outcome corresponds with current studies, since physical and mental functioning, as well as quality of life, have been found to decrease with an increase in the number of chronic conditions.^18^ Contrary to the study team’s hypothesis, negative illness perceptions and high mental preoccupation with illness cause did not correlate with pro-aging.

SOC showed significant correlation with low mental preoccupation and positive illness perceptions, especially concerning PS internal theories. Therefore, SOC seems to play an important role in the illness perception of people with obesity. SOC can be divided into three main components: comprehensibility (such as health perception, comprehension of health/illness, body image), meaningfulness (instrumental and absolute value of health), and manageability. Manageability can be further divided into the two groups, namely, internal resources (self-image, attitude, and self-help) and external resources (such as professional support, family coherence, work, and leisure time).^19^ Associations have been shown between strong SOC, an active lifestyle and increased health-related quality of life, less mental preoccupation, and less anxiety and depression in populations with cardiovascular risk.^20–21^ Strengthening SOC therefore could reduce PS effects of obesity or other illnesses aggravated by unfavorable illness perceptions. This appears to be a useful approach, as illness perception can be positively influenced and is not unchangeable.^22^

Study participants with high mental occupation and unfavorable illness perceptions were more likely to have pathological eating behavior, especially people with PS theories on the cause of obesity. Higher levels of mental occupation with overweight have been associated with disrupted cognitive patterns, leading to dysfunctional eating behavior which was also observed in both measurements of this study cohort. Several of the distinct cognitive patterns that have been shown to regulate eating and movement behavior are part of the AD-EVA assessment and were therefore investigated in this study.^23–24^ The summation of these patterns determines whether salutogenic behavior outweighs pathogenic eating behavior. As shown in this study cohort, PS theories seem to play an important role in the development of the abovementioned cognitive patterns and therefore of people’s eating behavior. This might be explained by the fact that PS problems are associated with the use of more emotion-based coping strategies (such as isolation, resignation, protest, and intrusion) and less problem-oriented strategies (such as minimization).**^25^** Mental preoccupation with illness causes and unfavorable illness perceptions were also associated with low emotional and physical well-being in this study, which is fitting, since more positive illness perceptions and better-perceived health correlate with better well-being and physical function.^26^

Overall mental occupation with overweight and obesity was high and participants seemed to be aware of the negative impact of obesity on health and possible complications. Therefore, conditions appear to be good for a successful intervention to establish healthy behavior and achieve weight loss. Nevertheless, the majority of subjects had tried at least one measure against overweight, with all attempts being unsuccessful or not effective in the long term. This study group feels confident that knowing a person’s theories on illness cause may be vital for a successful weight loss intervention, since a person’s way of coping with a disease and its consequences is influenced by and influences how an illness is perceived.**^27^** For example, a person who considers HB to be the main cause of their obesity might be successful participating in a weight-loss program focusing on caloric restriction and exercise. Whereas for a person with mainly PS theories, psychotherapy might be a better fit, since it is the right tool to treat PS problems such as depression, low self-esteem, and feelings of helplessness. Although HB was the most commonly represented theory for illness cause, neither PS nor NT theories can be neglected. Because theories on illness cause are so diverse and many participants had diffuse theories, simultaneous implementation of different treatment options should be considered instead of focusing on just one. As an example, the combination of lifestyle interventions such as diet and exercise with behavioral and/or psychological interventions has been proven to contribute to long-term weight loss by positively influencing cognitive restraint and control over binge eating and overeating.^28^

### Strengths and limitations

Illness perception and theories in people with overweight and obesity have barely been analyzed before, even though the epidemic burden of overweight and obesity is well known and professional support for changing illness perceptions has been proven in other studies to impact disease outcomes in a positive way.^4^ Moreover, this study has the advantage of being a follow-up study, meaning that people’s evolution between measurements could be investigated.

However, sample size was quite small and evolution of BFA between measurements could only be assessed for 87.5% of subjects (due to age <35 years at first measurement, thus not applicable for BFA calculation). Social activity scores showed massive deterioration, even though participants reported not being aware of any severe changes and other emotional–social parameters of BFA remained stable, indicating incorrect measurement or flawed calculation of scores. Results might have been affected by the COVID-19 pandemic, especially PS factors such as anxiety, stress, and quality of life. Some correlations were found between PATEF, AD-EVA, and BFA. Further investigations are needed to fully understand the interactions between illness perception, eating and movement behavior, and aging.

## Conclusion

Overweight and obesity increase the risk of developing several NCD. Despite great effort to reduce the prevalence of obesity, the numbers are still increasing. In this study population, mental occupation with cause of overweight and obesity was high, with HB and PS factors the predominant theories for being overweight. Higher levels of mental occupation were associated with disrupted cognitive patterns causing dysfunctional eating behavior. Strong SOC correlated with better subjective health and less mental preoccupation.

Therefore, strengthening of the SOC might be a promising approach to reduce PS effects of obesity, especially in the presence of unfavorable illness perceptions. The implementation of illness perceptions into treatment of obesity may improve long-term outcomes, since patients could be prescribed weight-loss interventions tailored to their needs.

## Ethical Approval and Informed Consent

Written informed consent was obtained from each participant. The study protocol was approved by the Cantonal Ethics Committee Bern (Ref.-Nr. KEK-BE: 2019–01109) and was in accordance with the 1964 Helsinki declaration and its later amendments or comparable ethical standards.

## Abbreviations Used

AD-EVA: Assessment of Eating and Movement Behaviour
AHA: Active and Healthy Aging
BFA: Bio-functional Age
BFAI: Bio-functional Age Index
BFS: Bio-functional Status
BMI: Body Mass Index
CA: Chronological Age
FBEB: Clinical Eating Disorder
FBM: Movement Motivation
FEV-Path: Pathogenic Eating Behaviour
FEV-Salute: Salutogenic Eating Behaviour
FUN: Handling of Food
FVE-PWS: Preclinical Eating Disorder
HADS: Hospital Anxiety and Depression Scale
HB: Health Behaviour
ICF: International Classification of Functioning, Disability and Health
NCD: Non-communicable Diseases
NT: Naturalistic
NTE: Naturalistic External
NTI: Naturalistic Internal
OS: Overall Score
PATEF: Patient Theory Questionnaire
PS: Psychosocial
PSE: Psychosocial External
PSI: Psychosocial Internal
QOL: Quality of Life
SF-36: Short Form (36) Health Survey
SKB: Body Image
SOC: Sense of Coherence
SPSS: Statistical Package for the Social Sciences
TICS: Trier Inventory for Chronic Stress
