# Supplementary material for: Impact of Illness Perception in Overweight and Obesity on Bio-Functional Age and Eating/Movement Behavior—A Follow-Up Study
Source: Womens Health Rep (New Rochelle). 2024 Oct 10;5(1):794–804. doi: 10.1089/whr.2024.0012 (PMC11491568; doi:10.1089/whr.2024.0012)
Supplement: Supplementary Data S1 [file whr.2024.0012_supp_datas1.pdf]

Supplementary File 1: Exemplification of bio-functional status (BFS) and bio-functional age (BFA)

Physical parameters

	VALUE		UNFAVOURABLE – FAVOURABLE
Systolic blood pressure*	117,00	mmHg	
Diastolic blood pressure	90,00	mmHg	
Pulse performance index	2,58		
Pulse rate difference	62,00	n / min	
Exercise heart rate	122,00	n / min	
Performance time	23,99	sec	
Vital capacity	73,35	%	
Hand grip strength	56,40	kp	
Fat mass*	10,85	kg	
Active cell mass	22,36	kg	
Teeth status - decayed, missing, filled	10,00	n	

* bipolar

strengths age class average resources

Sensory physiology and psychomotor parameters

	VALUE		UNFAVOURABLE – FAVOURABLE
Vision right	83,00	%	
Vision left	83,00	%	
Hearing loss right 2048 Hz	2,90	%	
Hearing loss right 4096 Hz	0,10	%	
Hearing loss left 2048 Hz	0,20	%	
Hearing loss left 4096 Hz	0,05	%	
Start rate	3,10	Hz	
Test motivation	2,60	Hz	
Psychomotor endurance	4,70	Hz	
Visuomotor coordination ability / time	104,61	sec	
Visuomotor coordination ability / mistakes	1,00	n	

strengths age class average resources

Cognitive and mental parameters

	VALUE		UNFAVOURABLE	FAVOURABLE
Optical reaction time	263,40	msec		
Acoustical reaction time	281,80	msec		
Pursuing reaction time	32,10	msec		
Verbal reaction time	9,00	sec		
Cognitive reaction time	12,00	sec		
Cognitive switching capability	19,00	sec		
Ability to concentrate / time	106,00	sec		
Ability to concentrate / mistakes	2,00	n		
Strategic thinking	81,43	sec		
Memory performance	98,00	n		
Orientation capability	44,00	n		
Change over capability	0,57	n		

strengths age class average resources

Emotional-social parameters

	VALUE	UNFAVOURABLE	FAVOURABLE
Physical wellbeing	11,00		
Emotional wellbeing	9,00		
Sense of coherence	47,00		
Stress exposition	36,00		
Social dominance*	19,00		
Social power	29,00		
Stress predisposition	64,00		
Social activity / duties	29,00		
Social activity / leisure	10,00		

* bipolar

strengths age class average resources

Transformation from BFS to BFA

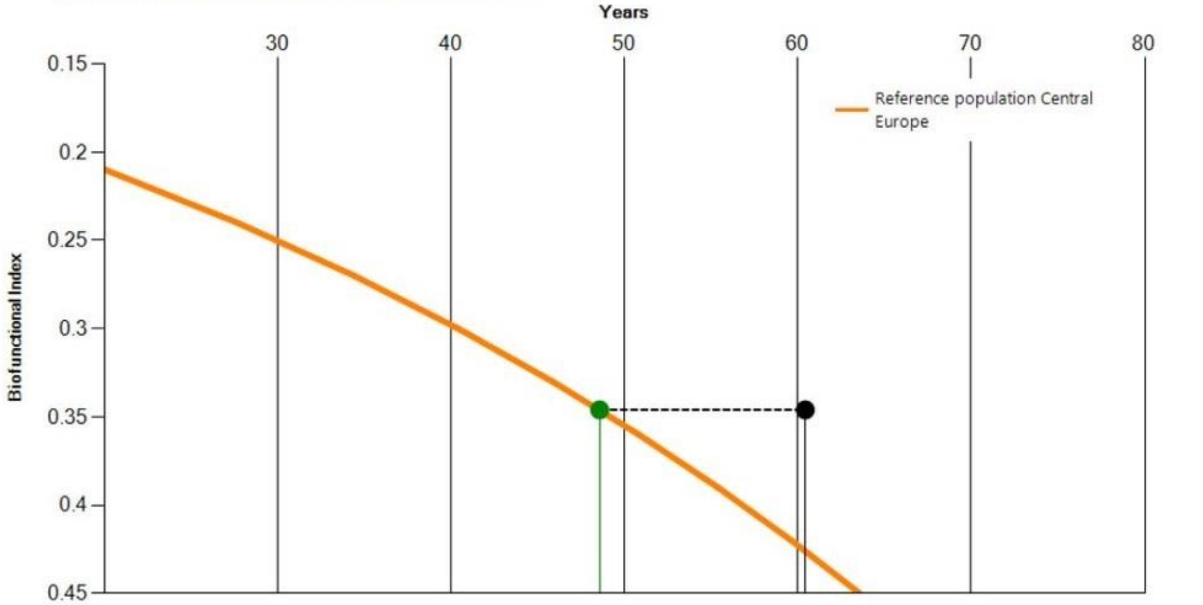

Chronological age (years)	60.43
Your Biofunctional age index (BFA)	48.57
