# Supplementary material for: Impact of Illness Perception in Overweight and Obesity on Bio-Functional Age and Eating/Movement Behavior—A Follow-Up Study
Source: Womens Health Rep (New Rochelle). 2024 Oct 10;5(1):794–804. doi: 10.1089/whr.2024.0012 (PMC11491568; doi:10.1089/whr.2024.0012)
Supplement: Supplementary Data S2 [file whr.2024.0012_supp_datas2.pdf]

Supplementary File 2: All results (A: BFA, B: AD-EVA, C: PATEF)

Table A: BFA (bio-functional age) – comparison between second (M02) and first (M01) measurement

	M02		M01		Difference mean value M02-M01	Difference statistically relevant (p<0.05)
	Mean (SD)	Range (min-max)	Mean (SD)	Range (min-max)		
Age parameters						
Chronological age [years]	55.0 (11.4)	27.8 - 72.1	49.2 (11.3)	22.3 - 66.3	5.8	Z=-5.511, p=0.000, r=0.6161
Bio-functional age [years]	53.9 (8.0)	37.9 - 67.1	44.4 (8.1)	27.8 - 65.8	9.5	Z=-5.069, p=0.000, r=0.6147
Difference chronological vs. bio-functional age (= chronological age – bio-functional age) [years]	4.3 (6.9)	-10.7 - 23.7	8.7 (8.5)	-8.0 - 26.7	-4.4	Z=-3.582, p=0.000, r=0.4344
Physical parameters						
Body height [cm]	167.3 (8.2)	152.0 - 186.0	168.0 (8.2)	153.0 - 187.0	-0.7	Z=-3.088, p=0.002, r=0.3496
Body weight [kg]	85.0 (17.7)	54.0 - 137.0	85.2 (15.4)	64.0 - 137.0	-0.2	
Systolic blood pressure [mmHg]	136.0 (15.7)	113.0 - 185.0	131.8 (17.2)	107.0 - 169.0	4.2	Z=-2.248, p=0.025, r=0.2513
Diastolic blood pressure [mmHg]	86.8 (9.6)	71.0 - 109.0	83.6 (10.9)	67.0 - 107.0	3.2	
Resting heart rate (p0) [n/min]	66.9 (8.9)	55.0 - 91.0	70.8 (10.2)	49.0 - 102.0	-3.9	Z=-2.091, p=0.037, r=0.2464
Exercise heart rate [n/min]	118.9 (19.3)	72.0 - 160.0	125.3 (14.4)	94.0 - 152.0	-6.4	Z=-2.279, p=0.023, r=0.2686
Pulse rate difference (Δp) [n/min]	52.0 (20.3)	9.0 - 92.0	54.7 (14.2)	26.0 - 83.0	-2.7	
Performance time [sec]	28.5 (6.8)	18.8 - 48.9	26.5 (5.3)	17.0 - 44.0	-2.0	Z=-2.498, p=0.012, r=0.2944
Pulse performance index (PPI = Δp/performance time)	1.9 (0.9)	0.3 - 3.9	2.1 (0.7)	1.1 - 4.7	-0.2	Z=-2.066, p=0.039, r=0.2435
Vital capacity [%]	97.1 (15.3)	62.8 - 132.8	97.7 (20.6)	56.6 - 127.2	-0.6	
Hand grip strength, both sides [kp]	57.8 (19.9)	29.0 - 123.5	60.9 (29.5)	18.0 - 140.0	-3.1	
Fat mass [kg]	32.8 (10.6)	10.9 - 51.9	35.0 (7.9)	23.1 - 49.6	-2.2	
Fat mass [%]	37.9 (7.4)	20.1 - 49.3	41.3 (6.3)	29.2 - 59.5	-3.4	Z=-2.293, p=0.022, r=0.2741
Active cell mass [kg]	27.3 (6.0)	19.2 - 50.0	28.2 (7.3)	11.9 - 60.0	-0.9	
Active cell mass [%]	32.5 (4.6)	24.4 - 42.4	33.5 (7.3)	15.2 - 65.2	-1.0	
Teeth status – decayed, missing, or filled teeth [n]	15.0 (6.3)	4.0 - 32.0	13.2 (6.8)	1.0 - 32.0	1.8	Z=-2.657, p=0.008, r=0.3008
Sensory physiology and psychomotor parameters						
Vision right [%]	60.4 (29.5)	16.0 - 100.0	87.1 (22.9)	42.0 - 100.0	-26.7	Z=-2.113, p=0.035, r=0.4313
Vision left [%]	59.7 (31.6)	16.0 - 100.0	89.8 (18.1)	50.0 - 100.0	-30.1	Z=-2.807, p=0.005, r=0.5730
Hearing loss right 2048 Hz [%]	5.5 (6.3)	0.4 - 22.4	4.5 (5.4)	0.2 - 22.4	1.0	

Hearing loss right 4096 Hz [%]	2.3 (2.4)	0.0 - 8.0	1.5 (2.1)	0.0 - 9.7	0.8	Z=-2.835, p=0.005, r=0.3252
Hearing loss left 2048 Hz [%]	5.2 (7.1)	0.2 - 28.0	3.4 (4.0)	0.2 - 17.3	1.8	Z=-2.187, p=0.029, r=0.2476
Hearing loss left 4096 Hz [%]	2.6 (3.6)	0.1 - 14.6	1.8 (2.4)	0.0 - 9.7	0.8	Z=-2.029, p=0.042, r=0.2297
Start rate (tapping frequency part 1) [Hz]	5.5 (1.4)	2.3 - 7.5	6.3 (0.6)	5.1 - 7.6	-0.8	Z=-3.271, p=0.001, r=0.3855
Test motivation (tapping frequency part 2) [Hz]	0.9 (0.6)	0.0 - 2.6	5.7 (0.5)	4.7 - 7.4	-4.8	Z=-5.233, p=0.000, r=0.6167
Psychomotor endurance (tapping frequency part 3) [Hz]	4.9 (0.9)	2.7 - 6.3	5.7 (0.6)	4.6 - 7.4	-0.8	Z=-4.598, p=0.000, r=0.5419
Visuomotor coordination ability (time) [sec]	35.6 (19.1)	13.1 - 104.6	35.0 (12.7)	12.6 - 61.3	0.6	
Visuomotor coordination ability (mistakes) [n]	8.6 (4.4)	1.0 - 21.0	11.0 (5.1)	1.0 - 24.0	-2.4	Z=-2.080, p=0.038, r=0.2326
Cognitive and mental parameters						
Optical reaction time [msec]	312.0 (35.8)	243.6 - 383.9	284.3 (40.2)	198.0 - 374.0	27.7	Z=-2.972, p=0.003, r=0.3365
Acoustical reaction time [msec]	276.3 (36.6)	203.1 - 336.9	306.2 (56.7)	209.0 - 420.0	-29.9	Z=-2.805, p=0.005, r=0.3176
Pursuing reaction time [msec]	51.8 (21.8)	24.7 - 127.0	75.2 (27.6)	21.0 - 156.0	-23.4	Z=-4.772, p=0.000, r=0.5335
Verbal reaction time [sec]	12.4 (1.7)	8.0 - 16.0	10.8 (1.3)	8.0 - 13.0	1.6	Z=-4.431, p=0.000, r=0.4954
Cognitive reaction time [sec]	14.2 (2.5)	10.0 - 23.0	12.9 (1.9)	10.0 - 19.0	1.3	Z=-3.776, p=0.000, r=0.4222
Cognitive switching capability [sec]	27.4 (6.8)	16.0 - 43.0	23.9 (4.9)	14.0 - 33.0	3.5	Z=-3.925, p=0.000, r=0.4388
Ability to concentrate (time) [sec]	124.7 (39.0)	58.0 - 211.0	130.6 (33.0)	64.0 - 195.0	-5.9	
Ability to concentrate (mistakes) [n]	2.4 (3.0)	0.0 - 14.0	2.1 (2.4)	0.0 - 10.0	0.3	
Strategic thinking [msec]	198.0 (131.3)	81.4 - 835.3	155.8 (67.5)	85.7 - 419.3	42.2	Z=-2.030, p=0.042, r=0.2270
Memory performance [n]	108.9 (32.5)	83.0 - 238.0	97.3 (28.6)	69.0 - 228.0	11.6	Z=-2.897, p=0.004, r=0.3239
Orientation capability [n]	56.0 (16.6)	34.0 - 101.0	48.5 (17.0)	29.0 - 121.0	7.5	Z=-2.264. p=0.024, r=0.2531
Change over capability [sec]	1.2 (0.7)	0.6 - 4.5	1.1 (0.2)	0.6 - 1.5	0.1	
Emotional-social parameters						
Physical wellbeing [score]	4.0 (4.6)	0.0 - 21.0	4.4 (4.8)	0.0 - 18.0	-0.4	
Emotional wellbeing [score]	1.2 (2.3)	0.0 - 9.0	1.3 (1.7)	0.0 - 6.0	-0.1	
Overall physical and emotional wellbeing [score]	5.2 (6.4)	0.0 - 30.0	5.7 (5.9)	0.0 - 23.0	-0.5	
Sense of coherence [score]	53.5 (6.6)	34.0 - 63.0	52.4 (7.6)	30.0 - 63.0	1.1	
Stress exposition [score]	50.8 (10.9)	25.0 - 69.0	53.3 (9.3)	28.0 - 69.0	-2.5	
Social dominance [score]	49.3 (11.3)	19.0 - 64.0	50.2 (8.7)	25.0 - 66.0	-0.9	
Social power [score]	39.2 (7.4)	26.0 - 55.0	39.4 (8.5)	26.0 - 59.0	-0.2	
Stress predisposition [score]	47.3 (8.9)	28.0 - 65.0	46.9 (9.9)	24.0 - 62.0	0.4	
Social activity / duties [score]	51.1 (14.1)	26.0 - 79.0	93.2 (11.3)	66.0 - 118.5	-42.1	Z=-5.512, p=0.000, r=0.6163
Social activity / leisure [score]	34.1 (11.6)	10.0 - 51.0	53.7 (12.8)	29.5 - 79.0	-19.6	Z=-5.202, p=0.000, r=0.5816

Table B: Prevalence of various eating and movement behaviour patterns AD-EVA – comparison between second (M02) and first (M01) measurement

	T-Values M02		T-Values M01		Difference mean value M02-M01	Difference statistically relevant (p<0.05)
	Mean (SD)	Range (min-max)	Mean (SD)	Range (min-max)		
Questionnaire						
Salutogenic eating behaviour - Overall score (FEV-Salute-GES)	54.6 (10.8)	31 - 75	52.8 (10.9)	36 - 80	1.8	
Salutogenic eating behaviour - Sports (FEV-Salute-S)	53.3 (9.9)	32 - 75	51.8 (10.4)	35 - 75	1.5	
Salutogenic eating behaviour - Mean control (FEV-Salute-MK)	50.9 (11.4)	21 - 76	48.4 (11.0)	34 - 80	2.5	
Salutogenic eating behaviour - Ability to enjoy (FEV-Salute-GE)	55.3 (10.0)	35 - 70	52.9 (9.9)	35 - 75	2.4	
Salutogenic eating behaviour - Ability to implement suggestions (FEV-Salute-EU)	54.9 (8.0)	42 - 77	56.6 (7.3)	43 - 77	-1.7	
Pathogenic eating behaviour - Cognitive control (FEV-Path-K)	52.4 (7.7)	36 - 69	55.0 (8.4)	39 - 72	-2.6	
Pathogenic eating behaviour - Disinhibition (FEV-Path-S)	45.0 (6.9)	31 - 60	48.2 (8.2)	31 - 74	-3.2	Z=-2.816, p=0.005, r=0.3148
Pathogenic eating behaviour - Emotional eating (FEV-Path-EE)	50.6 (9.3)	35 - 74	52.2 (8.5)	38 - 72	-1.6	
Handling of food (FUN)	43.6 (10.3)	27 - 75	45.3 (10.4)	31 - 65	-1.7	
Pre-clinical eating disorder (FVE)	46.8 (11.9)	20 - 80	48.2 (11.7)	27 - 68	-1.4	
Clinical eating disorder - Overall score binge eating disorder (FBEB-BED)	42.7 (8.9)	29 - 71	47.9 (11.8)	35 - 74	-5.2	Z=-2.960, p=0.003, r=0.3309
Clinical eating disorder - Overall score bulimia (FBEB-BUL)	43.8 (8.3)	29 - 74	48.8 (10.5)	35 - 76	-5.0	Z=-3.552, p=0.000, r=0.3971
Clinical eating disorder - Big-eating (FBEB-BIG)	41.0 (8.7)	33 - 69	44.4 (8.6)	33 - 67	-3.4	Z=-3.060, p=0.002, r=0.3421
Clinical eating disorder - Binge eating and compensation (FBEB-BuK)	48.6 (5.5)	41 - 72	51.0 (8.2)	41 - 76	-2.4	Z=-4.858, p=0.000, r=0.5431
Quality of life (SLQ)	54.2 (8.5)	21 - 69	44.6 (15.4)	24 - 72	9.6	Z=-3.268, p=0.001, r=0.3654
Movement motivation - Overall score (FBM-GES)	41.6 (6.6)	31 - 58	41.1 (7.1)	28 - 63	0.5	
Movement motivation - Fun and satisfaction (FBM-S&B)	38.7 (6.1)	31 - 55	38.5 (7.6)	25 - 67	0.2	
Movement motivation - Aesthetics (FBM-Ä)	51.7 (8.1)	34 - 68	51.2 (8.0)	30 - 68	0.5	
Dietary preferences - Overall score (EPL-GES)	53.2 (8.2)	36 - 71	54.9 (6.4)	45 - 68	-1.7	
Dietary preferences - Healthy food (EPL-G)	62.2 (7.7)	49 - 80	60.9 (9.7)	35 - 79	1.3	
Dietary preferences - Hearty food (EPL-D)	52.2 (11.7)	32 - 80	53.4 (11.4)	24 - 71	-1.2	
Dietary preferences - Snacks (EPL-S)	49.3 (8.4)	32 - 62	51.6 (6.9)	34 - 62	-2.3	
Socioeconomic group (1 – highest, 5 – lowest)	1.9 (1.1)	1 - 4	1.9 (1.1)	1 - 5	0	-
BMI group (1 – lowest, 6 – highest)	3.6 (1.1)	1 - 6	3.6 (0.8)	2 - 6	0	-
BMI (in kg/m^2)	30.4 (5.0)	18.0 - 44.2	30.2 (4.5)	21.7 - 44.2	0.2	-
Lowest weight (in kg)	64.9 (12.5)	46 - 100	65.0 (14.6)	20 - 100	-0.1	-
Highest weight (in kg)	91.6 (16.5)	65 - 145	89.5 (14.8)	65 - 125	2.1	-

		Values M02		Values M01		Difference mean value M02-M01	Difference statistically relevant (p<0.05)
		Mean (SD)	Range (min-max)	Mean (SD)	Range (min-max)		
Mother's weight (1 – lowest, 5 – highest)		2.9 (0.9)	1 - 5	2.9 (0.8)	1 - 4	0	-
Father's weight (1 – lowest, 5 – highest)		2.6 (0.8)	1 - 5	2.7 (0.9)	1 - 5	-0.1	-
Body image – self-perception (1 – lowest, 6 – highest)		3.7 (0.9)	2 - 6	3.7 (0.8)	2 - 5	0	-
Body image – aspired ideal (1 – lowest, 6 – highest)		2.6 (0.5)	2 - 3	2.6 (0.6)	2 - 4	0	-
Lifestyle – smoking (1 – lowest, 4 – highest)		1.4 (1.1)	1 - 4	3.0 (1.2)	1 - 5	-1.6	-
Lifestyle – alcohol (1 – lowest, 4 – highest)		2.4 (0.8)	1 - 4	2.3 (0.7)	1 - 4	0.1	-
Lifestyle – night eating (1 – lowest, 4 – highest)		1.3 (0.8)	1 - 4	1.2 (0.7)	1 - 4	0.1	-
		M02 [%]		M01 [%]			
Socioeconomic group	Group 1 (highest)	52.5		50			
	Group 2	20		17.5			
	Group 3	15		25			
	Group 4	12.5		5			
	Group 5 (lowest)	0		2.5			
BMI group	Underweight (BMI <18.5 kg/m^2)	2.5		0			
	Normal weight (BMI 18.5-24.9 kg/m^2)	7.5		2.5			
	Overweight (BMI 25-29.9 kg/m^2)	42.5		50			
	Obesity I (BMI 30-34.9 kg/m^2)	25		35			
	Obesity II (BMI 35-39.9 kg/m^2)	20		10			
	Obesity III (BMI ≥40 kg/m^2)	2.5		2.5			
Measures against overweight	None	20		27.5			
	Yes, at least one	80		72.5			
	Doctor consultation	27.5		27.5			
	Nutrition consultation	42.5		50			
	Physiotherapy, sports expert consultation	5		17.5			
	Diet	55		50			
	Stay at a health resort	0		2.5			
	Weight Watchers	25		22.5			
	Surgery	2.5		0			
Mother's weight	1 (very slim)	2.5		5			
	2 (slim)	32.5		25			
	3 (chubby)	45		47.5			
	4 (fat)	15		22.5			

	5 (very fat)	5	0
Father's weight	1 (very slim)	5	5
	2 (slim)	40	40
	3 (chubby)	40	40
	4 (fat)	7.5	12.5
	5 (very fat)	2.5	2.5
Body image – self-perception	1 (underweight)	0	0
	2 (very slim)	5	2.5
	3 (slim)	45	37.5
	4 (normal)	27.5	40
	5 (fat)	17.5	17.5
	6 (very fat)	2.5	0
Body image – aspired ideal	1 (underweight)	0	0
	2 (very slim)	42.5	47.5
	3 (slim)	57.5	45
	4 (normal)	0	7.5
	5 (fat)	0	0
	6 (very fat)	0	0
Discrepancy between scores of self-perception and BMI group: participants consider themselves…	-2 = much slimmer	0	5
	-1 = slimmer	25	15
	0 = weighing corresponding to BMI group	47.5	40
	1 = heavier	17.5	35
	2 = much heavier	5	2.5
	3 = extremely heavier	2.5	0
Lifestyle – smoking	Never	82.5	15
	1x per month	0	17.5
	1-2x per week	2.5	17.5
	Daily	12.5	45
Lifestyle – alcohol	Never	12.5	2.5
	1x per month	40	70
	1-2x per week	40	15
	Daily	5	7.5
Lifestyle – night eating	Never	82.5	87.5
	1x per month	7.5	2.5
	1-2x per week	2.5	5
	Daily	5	2.5

Table C1: PATEF – Distribution of mental occupation on subscales

Distribution of mental occupation on subscales of PATEF: upper value M02 (n=39), lower value M01 (n=40)									
Degree of mental occupa-tion	S-value	Subscales of PATEF							
		Over-all score (OS) [%]	Health beha-viour scale (HB) [%]	Naturalistic			Psychosocial		
				Overall natura-listic scale (NT) [%]	External scale (NTE) [%]	Internal scale (NTI) [%]	Overall psycho-social scale (PS) [%]	External scale (PSE) [%]	Internal scale (PSI) [%]
Low	1	-<br>-	-<br>-	-<br>-	-<br>-	-<br>-	-<br>-	-<br>-	-<br>-
	2	2.6<br>-	-<br>-	5.1<br>10.0	-<br>-	-<br>-	-<br>-	-<br>-	-<br>-
	3	5.1<br>7.5	2.6<br>-	2.6<br>5.0	15.4<br>30.0	12.8<br>15.0	7.7<br>5.0	-<br>-	-<br>-
Mode-rate	4	15.4<br>22.5	12.8<br>20.0	25.6<br>27.5	23.1<br>22.5	5.1<br>7.5	2.6<br>12.5	12.8<br>15.0	15.4<br>12.5
	5	25.6<br>15.0	20.5<br>10.0	10.3<br>30.0	20.5<br>27.5	25.6<br>32.5	15.4<br>15.0	10.3<br>22.5	17.9<br>22.5
	6	17.9<br>37.5	15.4<br>15.0	25.6<br>17.5	15.4<br>17.5	35.9<br>32.5	30.8<br>25.0	35.9<br>30.0	23.1<br>12.5
High	7	23.1<br>10.0	15.4<br>20.0	20.5<br>5.0	20.5<br>2.5	7.7<br>7.5	28.2<br>35.0	33.3<br>27.5	35.9<br>40.0
	8	2.6<br>2.5	20.5<br>22.5	7.7<br>5.0	5.1<br>-	12.8<br>2.5	12.8<br>5.0	5.1<br>2.5	2.6<br>5.0
	9	7.7<br>5.0	12.8<br>12.5	2.6<br>-	-<br>-	-<br>2.5	2.6<br>2.5	2.6<br>2.5	5.1<br>7.5

Table C2: PATEF – Degree of mental occupation

	M02 [%]	M01 [%]
No excessive thoughts or theory not covered	7.5	7.5
High mental occupation with illness cause	32.5	17.5
Mental occupation with illness cause, but indecisiveness about cause	25.0	22.5
Other	32.5	52.5

Table C3: PATEF – Diffuse theory vs. established theory

	M02 [%]	M01 [%]
Established theory	42.5	60.0
Diffuse theory	35.0	17.5
Other	20.0	22.5

Table C4: PATEF – Psychosocial vs. naturalistic theory

	M02 [%]	M01 [%]
Rejection of PS and NT	2.5	0.0
PS and NT unremarkable	37.5	37.5
PS and NT considered to be very likely	25.0	7.5
Focus clearly on PS	17.5	35.0
Focus clearly on NT	5.0	2.5
Other	10.0	17.5

Table C5: PATEF group analysis (grouped by sex, age, grade of aging, and BMI group) - comparison between second measurement (M02, upper value) and first measurement (M01, lower value) in mean values

	T-value of PATEF subscales: Mean (SD)									
	PSE	PSI	HB	NTE	NTI	PS	NT	OS	S-values >5	S-values >6
Grouped by sex										
Female (n=34)	6.12 (1.11)<br>5.88 (1.23)	6.00 (1.32)<br>6.18 (1.42)	6.48 (1.79)<br>6.50 (1.75)	5.06 (1.56)<br>4.38 (1.18)	5.64 (1.43)<br>5.26 (1.40)	6.18 (1.45)<br>5.97 (1.45)	5.52 (1.70)<br>4.74 (1.48)	5.70 (1.69)<br>5.47 (1.52)	4.79 (2.88)<br>4.06 (3.05)	2.91 (2.99)<br>2.26 (2.30)
Male (n=6)	6.33 (1.63)<br>5.83 (1.17)	6.50 (1.38)<br>6.67 (1.37)	6.00 (1.41)<br>6.67 (1.63)	5.83 (1.17)<br>4.50 (1.23)	5.33 (1.63)<br>5.33 (1.37)	6.17 (1.33)<br>6.00 (1.10)	5.67 (1.63)<br>4.83 (1.60)	5.67 (1.63)<br>5.50 (1.23)	5.33 (2.50)<br>4.50 (2.74)	2.67 (3.14)<br>2.33 (1.75)
Grouped by age										
<45 years (n=7)	6.71 (0.95)<br>6.14 (0.69)	6.43 (1.51)<br>6.71 (1.25)	7.43 (1.40)<br>7.57 (1.40)	5.71 (1.80)<br>5.43 (0.79)	6.57 (1.40)<br>5.71 (0.95)	6.71 (1.11)<br>6.43 (0.98)	6.57 (1.90)<br>5.57 (1.13)	6.71 (1.89)<br>6.29 (1.25)	5.86 (3.13)<br>6.29 (2.56)	4.86 (3.39)<br>2.86 (2.19)
≥45 years (n=33)	6.03 (1.20)<br>5.82 (1.29)	6.00 (1.30)<br>6.15 (1.44)	6.19 (1.73)<br>6.30 (1.70)	5.06 (1.46)<br>4.18 (1.13)	5.38 (1.39)<br>5.18 (1.45)	6.06 (1.46)<br>5.88 (1.45)	5.31 (1.55)<br>4.58 (1.50)	5.47 (1.55)<br>5.30 (1.47)	4.66 (2.73)<br>3.67 (2.88)	2.44 (2.75)<br>2.15 (2.22)
Grouped by grade of aging										
Pro-aging (n=4)	5.00 (1.00)<br>5.25 (1.26)	5.33 (1.53)<br>5.75 (1.50)	4.33 (0.58)<br>6.25 (1.71)	4.00 (1.00)<br>4.00 (0.82)	4.67 (1.53)<br>5.00 (0.82)	5.00 (2.00)<br>5.25 (1.71)	4.00 (2.00)<br>4.74 (0.50)	4.00 (2.00)<br>5.00 (1.41)	2.00 (3.46)<br>2.75 (2.63)	0.67 (1.16)<br>1.50 (1.29)
Physiologic aging (n=22)	6.09 (1.19)<br>5.64 (1.33)	5.86 (1.39)<br>6.00 (1.48)	6.18 (1.79)<br>5.95 (1.73)	5.27 (1.42)<br>4.00 (1.11)	5.32 (1.49)<br>4.95 (1.36)	5.95 (1.46)<br>5.68 (1.49)	5.45 (1.57)<br>4.27 (1.52)	5.45 (1.57)<br>5.05 (1.40)	4.68 (2.72)<br>3.09 (2.81)	2.32 (2.61)<br>1.68 (2.01)
Healthy slow aging (n=8)	6.25 (1.17)<br>6.63 (0.74)	6.50 (0.76)<br>6.88 (1.13)	7.00 (1.20)<br>7.38 (1.19)	5.00 (1.60)<br>5.00 (1.20)	5.88 (0.84)<br>6.00 (1.69)	6.75 (0.89)<br>6.88 (0.64)	5.63 (1.30)<br>5.50 (1.51)	6.13 (0.84)<br>6.25 (1.39)	5.88 (1.89)<br>6.25 (2.19)	3.38 (3.20)<br>3.88 (2.36)
Grouped by BMI group										
Group 1: BMI <19.0 kg/m^2 (n=1)	7.00 (0.00)<br>7.00 (0.00)	9.00 (0.00)<br>9.00 (0.00)	9.00 (0.00)<br>9.00 (0.00)	5.00 (0.00)<br>4.00 (0.00)	8.00 (0.00)<br>5.00 (0.00)	8.00 (0.00)<br>8.00 (0.00)	7.00 (0.00)<br>4.00 (0.00)	9.00 (0.00)<br>8.00 (0.00)	7.00 (0.00)<br>5.00 (0.00)	7.00 (0.00)<br>5.00 (0.00)
Group 2: BMI 19.0-24.9 kg/m^2 (n=3)	4.50 (0.71)<br>5.00 (1.73)	5.00 (1.41)<br>5.33 (1.53)	4.50 (0.71)<br>4.67 (1.16)	4.00 (1.41)<br>3.67 (1.16)	4.50 (2.12)<br>4.00 (1.00)	4.50 (2.12)<br>4.67 (2.08)	3.50 (2.12)<br>3.67 (1.53)	3.50 (2.12)<br>4.00 (1.73)	1.50 (2.12)<br>1.67 (2.89)	0.00 (0.00)<br>1.00 (1.73)
Group 3: BMI 25.0-29.9 kg/m^2 (n=17)	6.12 (1.05)<br>5.71 (1.21)	5.82 (1.07)<br>6.06 (1.03)	6.18 (1.43)<br>6.06 (1.64)	5.24 (1.56)<br>4.35 (1.37)	5.35 (1.54)<br>5.24 (1.44)	6.06 (0.97)<br>5.88 (1.11)	5.41 (1.87)<br>4.53 (1.70)	5.47 (1.33)<br>5.24 (1.20)	4.71 (2.39)<br>4.18 (3.07)	2.29 (2.87)<br>1.88 (2.09)
Group 4: BMI 30.0-34.9 kg/m^2 (n=10)	6.10 (1.20)<br>6.20 (1.23)	6.10 (1.60)<br>5.90 (1.60)	6.50 (2.17)<br>7.10 (1.45)	5.40 (1.71)<br>4.40 (1.08)	5.80 (1.32)<br>5.30 (1.06)	6.20 (1.75)<br>6.00 (1.49)	5.80 (1.48)<br>4.90 (1.10)	5.80 (1.81)<br>5.60 (1.51)	4.80 (3.58)<br>3.70 (3.13)	3.30 (3.23)<br>2.40 (2.46)
Group 5: BMI 35.0-39.9 kg/m^2 (n=8)	6.50 (1.41)<br>6.00 (1.07)	6.38 (1.19)<br>7.00 (1.51)	6.88 (1.73)<br>7.00 (1.77)	4.88 (1.36)<br>4.63 (0.92)	5.50 (0.93)<br>5.63 (1.69)	6.50 (1.60)<br>6.38 (1.51)	5.50 (1.07)<br>5.25 (1.28)	6.00 (1.60)<br>5.88 (1.55)	5.50 (2.56)<br>4.88 (2.64)	3.13 (2.48)<br>2.75 (2.25)
Group 6: BMI ≥40.0 kg/m^2 (n=1)	7.00 (0.00)<br>6.00 (0.00)	7.00 (0.00)<br>7.00 (0.00)	7.00 (0.00)<br>8.00 (0.00)	7.00 (0.00)<br>6.00 (0.00)	8.00 (0.00)<br>7.00 (0.00)	7.00 (0.00)<br>6.00 (0.00)	8.00 (0.00)<br>7.00 (0.00)	7.00 (0.00)<br>7.00 (0.00)	8.00 (0.00)<br>8.00 (0.00)	8.00 (0.00)<br>5.00 (0.00)

Table C6: PATEF group analysis (grouped by sex, age, grade of aging, and BMI group) - comparison between second measurement (M02, upper value) and first measurement (M01, lower value) in percentages

	Overall mental preoccupation: percentage of the group with this degree of mental occupation with illness cause		
	Low (T-value 1-3)	Moderate (T-value 4-6)	High (T-value 7-9)
Grouped by sex			
Female (n=34)	7.2% (n=19)<br>9.2% (n=25)	56.4% (n=149)<br>62.9% (n=171)	36.4% (n=96)<br>27.9% (n=76)
Male (n=6)	4.2% (n=2)<br>8.3% (n=4)	62.5% (n=30)<br>62.5% (n=30)	33.3% (n=16)<br>29.2% (n=14)
Grouped by age			
<45 years (n=7)	1.8% (n=1)<br>0.0% (n=0)	37.5% (n=21)<br>64.3% (n=36)	60.7% (n=34)<br>35.7% (n=20)
≥45 years (n=33)	7.8% (n=20)<br>11.0% (n=29)	61.7% (n=158)<br>62.5% (n=165)	30.5% (n=78)<br>26.5% (n=70)
Grouped by grade of aging			
Pro-aging (n=4)	20.8% (n=5)<br>9.4% (n=3)	70.8% (n=17)<br>71.9% (n=23)	8.3% (n=2)<br>18.8% (n=6)
Physiologic aging (n=22)	7.4% (n=13)<br>13.6% (n=24)	63.6% (n=112)<br>65.9% (n=116)	29.0% (n=51)<br>20.5% (n=36)
Healthy slow aging (n=8)	3.1% (n=2)<br>3.1% (n=2)	54.7% (n=35)<br>48.4% (n=31)	42.2% (n=27)<br>48.4% (n=31)
Grouped by BMI group			
Group 1: BMI <19.0 kg/m^2 (n=1)	0.0% (n=0)<br>0.0% (n=0)	12.5% (n=1)<br>37.5% (n=3)	87.5% (n=7)<br>62.5% (n=5)
Group 2: BMI 19.0-24.9 kg/m^2 (n=3)	31.3% (n=5)<br>29.2% (n=7)	68.8% (n=11)<br>58.3% (n=14)	0.0% (n=0)<br>12.5% (n=3)
Group 3: BMI 25.0-29.9 kg/m^2 (n=17)	5.9% (n=8)<br>12.5% (n=17)	65.4% (n=89)<br>64.0% (n=87)	28.7% (n=39)<br>23.5% (n=32)
Group 4: BMI 30.0-34.9 kg/m^2 (n=10)	6.3% (n=5)<br>2.5% (n=2)	52.5% (n=42)<br>68.8% (n=55)	41.3% (n=33)<br>28.8% (n=23)
Group 5: BMI 35.0-39.9 kg/m^2 (n=8)	4.7% (n=3)<br>4.7% (n=3)	56.3% (n=36)<br>60.9% (n=39)	39.1% (n=25)<br>34.4% (n=22)
Group 6: BMI ≥40.0 kg/m^2 (n=1)	0.0% (n=0)<br>0.0% (n=0)	0.0% (n=0)<br>37.5% (n=3)	100% (n=8)<br>62.5% (n=5)
