# Supplementary material for: Impact of Illness Perception in Overweight and Obesity on Bio-Functional Age and Eating/Movement Behavior—A Follow-Up Study
Source: Womens Health Rep (New Rochelle). 2024 Oct 10;5(1):794–804. doi: 10.1089/whr.2024.0012 (PMC11491568; doi:10.1089/whr.2024.0012)
Supplement: Supplementary Data S3 [file whr.2024.0012_supp_datas3.pdf]

Supplementary File 3: All correlations

Table A: Correlations of PATEF (horizontal) with AD-EVA (vertical) – second measurement (M02)

	Overall-score (OS)	Psychosocial (PS)	Naturalistic (NT)	Psychosocial external (PSE)	Psychosocial internal (PSI)	Health behaviour (HB)	Naturalistic external (NTE)	Naturalistic internal (NTI)
EPL-D	-0.287	-0.182	-0.228	-0.115	-0.092	-0.282	-0.063	**-0.344***
EPL-G	-0.036	-0.114	0.136	0.106	-0.052	-0.155	0.227	-0.013
EPL-S	-0.199	-0.151	-0.276	-0.100	-0.102	-0.065	-0.228	**-0.328***
EPL-GES	-0.274	-0.227	-0.236	-0.129	-0.126	-0.191	-0.105	**-0.345***
FBEB-BED	0.054	0.075	-0.020	0.021	0.127	0.149	0.064	0.123
FBEB-BIG	0.066	0.068	0.011	0.046	0.172	0.160	0.158	0.051
FBEB-BuK	0.273	**0.334***	0.133	0.174	0.267	**0.349***	0.024	0.305
FBEB-BUL	0.103	0.113	0.089	0.036	0.131	0.185	0.121	0.223
FEV-Path-EE	**0.370***	**0.378***	**0.349***	**0.364***	**0.334***	**0.350***	0.226	**0.479****
FEV-Path-S	**0.387***	**0.486****	0.210	**0.419****	**0.437****	**0.342***	0.035	**0.419****
FEV-Path-K	**0.384***	0.285	**0.346***	**0.406***	0.219	0.313	0.193	**0.430****
FEV-Salute-MK	**-0.384***	**-0.499****	-0.227	**-0.412****	**-0.502****	**-0.474****	-0.245	**-0.320***
FEV-Salute-S	-0.221	-0.226	-0.121	-0.246	-0.200	**-0.375***	-0.205	-0.028
FEV-Salute-EU	0.150	0.041	0.195	0.047	-0.003	0.007	0.068	0.272
FEV-Salute-GE	-0.239	-0.106	**-0.334***	-0.128	-0.113	-0.195	**-0.331***	**-0.324***
FEV-Salute-GES	-0.274	**-0.326***	-0.179	-0.265	**-0.343***	**-0.397***	-0.255	-0.179
FUN	**0.468****	**0.488****	0.312	**0.343***	**0.528****	**0.399***	0.156	**0.559****
FVE	0.273	0.279	0.094	0.281	0.206	**0.434****	0.001	0.128
FBM-S&B	0.225	0.137	0.193	0.186	0.094	**0.355***	0.201	0.136
FBM-Ä	-0.282	-0.313	-0.231	**-0.354***	-0.216	-0.225	-0.057	**-0.346***
FBM-GES	0.039	-0.051	0.074	-0.009	-0.059	0.179	0.145	-0.015
SLQ	-0.272	-0.252	-0.080	-0.164	**-0.352***	-0.251	-0.063	-0.178

* = p < 0.05 / ** = p < 0.01

Table B: Correlations of PATEF (horizontal) with AD-EVA (vertical) – first measurement (M01)

	Overall-score (OS)	Psychosocial (PS)	Naturalistic (NT)	Psychosocial external (PSE)	Psychosocial internal (PSI)	Health behaviour (HB)	Naturalistic external (NTE)	Naturalistic internal (NTI)
EPL-D	-0.169	-0.018	-0.176	0.024	-0.009	-0.218	-0.236	-0.106
EPL-G	0.017	-0.062	0.086	0.138	-0.166	0.086	0.059	-0.129
EPL-S	0.093	0.192	-0.070	0.106	0.219	0.236	-0.053	-0.007
EPL-GES	0.075	0.138	-0.032	0.136	0.144	0.162	-0.078	-0.029
FBEB-BED	0.232	**0.521***	-0.081	0.340	0.291	0.062	0.031	-0.007
FBEB-BIG	0.188	0.241	0.053	0.201	0.185	0.113	0.100	0.048
FBEB-BuK	0.182	**0.499***	-0.242	0.367	0.254	0.060	-0.128	-0.041
FBEB-BUL	0.363	**0.590****	-0.027	**0.477***	0.370	0.175	0.054	0.076
FEV-Path-EE	0.195	**0.371***	-0.031	0.176	**0.377***	0.151	0.009	-0.044
FEV-Path-S	0.310	**0.349***	0.144	0.236	**0.335***	0.113	0.043	0.226
FEV-Path-K	-0.050	-0.032	0.045	0.007	-0.093	-0.044	0.167	-0.091
FEV-Salute-MK	**-0.434****	**-0.517****	-0.264	**-0.381***	**-0.541****	-0.302	-0.155	-0.303
FEV-Salute-S	-0.159	**-0.323***	0.081	-0.231	-0.259	-0.110	0.166	-0.067
FEV-Salute-EU	0.131	0.013	0.102	0.173	-0.043	0.122	-0.009	0.131
FEV-Salute-GE	-0.062	-0.061	-0.122	-0.080	-0.046	0.071	-0.140	-0.164
FEV-Salute-GES	-0.303	**-0.451****	-0.119	-0.312	**-0.443****	-0.172	-0.027	-0.182
FUN	**0.376***	**0.631****	0.118	**0.467****	**0.612****	0.126	0.010	0.153
FVE	0.156	0.104	0.243	0.148	-0.014	0.063	0.308	0.100
FBM-S&B	0.068	-0.006	0.172	-0.085	-0.004	0.102	0.161	0.138
FBM-Ä	-0.125	-0.047	-0.204	-0.087	-0.023	-0.020	-0.308	-0.097
FBM-GES	-0.003	0.016	-0.001	-0.067	0.024	0.086	-0.037	0.005
SLQ	0.157	-0.063	0.260	0.010	-0.025	0.305	**0.326***	0.152

* = $p < 0.05$ / ** = $p < 0.01$

Table C: Correlations of PATEF (horizontal) with BFA (vertical) – second measurement (M02)

	Overall-score (OS)	Psychosocial (PS)	Naturalistic (NT)	Psychosocial external (PSE)	Psychosocial internal (PSI)	Health behaviour (HB)	Naturalistic external (NTE)	Naturalistic internal (NTI)
BMI	0.290	0.255	0.268	0.258	0.158	0.193	0.160	0.225
Chronological age	-0.289	-0.255	-0.211	**-0.319***	-0.150	**-0.413****	-0.124	-0.210
Bio-functional age	0.083	0.083	0.193	0.109	0.152	-0.193	0.242	0.128
Difference (chron. – bio-f.)	-0.225	-0.220	-0.231	-0.245	-0.185	-0.143	-0.184	-0.249
Systolic blood pressure	-0.013	-0.114	0.164	0.026	-0.126	-0.217	0.215	0.023
Diastolic blood pressure	0.029	-0.086	0.003	0.044	-0.074	-0.081	-0.051	-0.014
Resting heart rate	**0.357***	0.241	**0.416***	0.224	0.151	0.176	**0.447****	0.190
Exercise heart rate	-0.200	-0.124	-0.152	-0.112	-0.192	-0.210	-0.033	-0.253
Pulse rate difference	-0.318	-0.194	-0.304	-0.171	-0.229	-0.246	-0.223	**-0.337***
Performance time	0.051	0.010	-0.072	-0.091	0.123	0.120	-0.133	-0.068
Pulse performance index	-0.276	-0.169	-0.185	-0.115	-0.274	-0.244	-0.075	-0.261
Vital capacity	0.191	0.263	0.107	**0.346***	0.129	0.160	0.104	0.152
Hand grip strength	-0.110	-0.139	-0.102	-0.056	0.012	-0.014	0.007	-0.208
Fat mass	0.248	0.217	0.207	0.199	0.096	0.212	0.009	0.262
Active cell mass	-0.150	-0.137	-0.072	-0.111	-0.084	-0.055	0.113	-0.235
Teeth status – DMF	-0.140	-0.154	-0.213	-0.147	-0.012	-0.217	-0.074	-0.220
Vision right	0.068	0.021	0.081	-0.152	-0.035	-0.077	-0.096	0.138
Vision left	0.369	0.208	0.437	0.006	0.264	0.355	0.152	0.506
Hearing loss right 2048 Hz	-0.230	-0.233	-0.184	-0.250	-0.154	-0.202	-0.092	-0.182
Hearing loss right 4096 Hz	-0.278	-0.177	-0.310	-0.220	-0.044	**-0.361***	-0.238	-0.175
Hearing loss left 2048 Hz	-0.093	-0.105	-0.113	-0.036	-0.086	-0.047	0.040	-0.222
Hearing loss left 4096 Hz	**-0.392***	-0.307	-0.290	-0.161	-0.283	**-0.519****	-0.115	**-0.344***
Start rate	**0.329***	**0.333***	0.171	**0.343***	0.246	**0.348***	0.115	0.137
Test motivation	**0.605****	**0.558****	**0.475****	**0.573****	**0.475****	**0.547****	0.299	**0.504****
Psychomotor endurance	0.288	0.322	0.125	0.311	0.264	**0.435****	0.089	0.060
Viseomotor (time)	0.117	0.136	-0.042	-0.018	0.136	0.222	-0.159	-0.044
Viseomotor (mistakes)	-0.237	-0.266	-0.199	**-0.352***	-0.141	-0.288	-0.203	-0.255
Optical reaction time	0.120	0.070	0.234	0.069	0.057	-0.024	0.275	0.198
Acoustical reaction time	-0.030	-0.153	0.096	-0.142	-0.147	-0.169	0.194	0.042

Pursuing reaction time	0.139	0.059	0.236	0.230	-0.100	-0.049	0.221	0.175
Verbal reaction time	-0.128	-0.109	0.005	-0.077	-0.111	-0.267	0.064	-0.104
Cognitive reaction time	-0.201	-0.130	-0.068	-0.168	-0.064	**-0.440****	-0.010	-0.114
Cognitive switching	0.069	0.139	0.057	0.127	0.172	-0.180	0.046	0.088
Concentration (time)	0.206	0.167	**0.333***	0.255	0.120	0.029	0.250	**0.473****
Concentration (mistakes)	-0.004	0.027	-0.110	-0.110	0.076	-0.029	-0.091	-0.070
Strategic thinking	-0.027	-0.036	0.125	-0.072	-0.160	-0.236	0.117	0.056
Memory performance	0.138	0.131	0.266	0.190	0.024	-0.133	0.269	0.174
Orientation capability	0.167	0.089	0.315	0.237	-0.051	-0.122	0.363	0.209
Change over capability	-0.018	-0.003	0.027	-0.120	-0.043	-0.110	-0.040	0.021
Physical wellbeing	**0.449****	**0.395***	0.292	0.248	**0.367***	**0.341***	0.119	**0.348***
Emotional wellbeing	**0.482****	**0.581****	0.299	**0.349***	**0.485****	**0.424****	0.094	**0.377***
Overall wellbeing	**0.510****	**0.479****	**0.323***	0.290	**0.433****	**0.412****	0.116	**0.389***
Sense of coherence	**-0.404***	**-0.529****	-0.207	**-0.368***	**-0.620****	**-0.364***	-0.098	**-0.366***
Stress exposition	-0.242	**-0.417****	-0.038	-0.256	**-0.413****	**-0.320***	-0.015	-0.157
Social dominance	-0.288	-0.113	-0.207	-0.190	-0.260	**-0.379***	**-0.324***	-0.285
Social power	-0.201	-0.053	-0.191	-0.084	-0.041	-0.128	-0.161	-0.220
Stress predisposition	0.128	-0.008	0.119	0.148	-0.024	0.069	0.008	0.264
Social activity / duties	-0.024	-0.054	-0.021	0.056	-0.171	-0.039	-0.140	-0.017
Social activity / leisure	-0.105	-0.001	-0.109	0.010	-0.038	-0.074	-0.162	0.015

* = $p < 0.05$ / ** = $p < 0.01$

Table D: Correlations of PATEF (horizontal) with BFA (vertical) – first measurement (M01)

	Overall-score (OS)	Psychosocial (PS)	Naturalistic (NT)	Psychosocial external (PSE)	Psychosocial internal (PSI)	Health behaviour (HB)	Naturalistic external (NTE)	Naturalistic internal (NTI)
BMI	**0.399***	0.289	**0.429****	0.304	0.308	**0.497****	**0.349***	**0.373***
Chronological age	**-0.316***	-0.142	-0.228	-0.219	-0.140	**-0.359***	-0.262	-0.296
Bio-functional age	0.150	0.157	0.278	0.173	0.135	0.105	0.316	0.129
Difference (chron. – bio-f.)	-0.282	-0.271	-0.329	-0.318	-0.213	-0.254	**-0.390***	-0.306
Systolic blood pressure	-0.280	-0.208	-0.170	-0.178	-0.124	-0.217	-0.120	-0.245
Diastolic blood pressure	**-0.344***	-0.183	-0.285	-0.156	-0.170	-0.253	-0.225	**-0.330***
Resting heart rate	0.201	0.111	0.287	0.093	0.105	0.306	0.225	0.279
Exercise heart rate	-0.260	**-0.372***	-0.032	**-0.428****	-0.300	-0.204	-0.058	-0.041
Pulse rate difference	**-0.341***	**-0.409****	-0.234	**-0.437****	**-0.337***	-0.287	-0.175	-0.222
Performance time	-0.108	0.043	-0.160	-0.010	0.031	-0.176	-0.144	-0.055
Pulse performance index	-0.210	**-0.338***	-0.114	**-0.317***	-0.258	-0.160	-0.105	-0.147
Vital capacity	0.249	0.304	0.156	0.282	**0.353***	0.292	0.195	0.115
Hand grip strength	-0.234	-0.283	-0.203	-0.205	-0.160	-0.154	-0.159	-0.101
Fat mass	0.174	0.222	0.168	0.062	0.185	0.171	**0.335***	0.005
Active cell mass	-0.297	-0.204	-0.221	-0.285	-0.212	**-0.449****	-0.213	-0.034
Teeth status – DMF	**-0.329***	-0.136	**-0.322***	-0.180	-0.168	**-0.324***	**-0.361***	-0.262
Vision right	0.269	0.463	-0.279	0.334	0.483	0.137	-0.181	-0.385
Vision left	0.148	0.381	-0.209	0.342	0.241	0.068	-0.205	-0.158
Hearing loss right 2048 Hz	-0.223	-0.182	-0.169	-0.141	-0.149	-0.138	-0.223	-0.163
Hearing loss right 4096 Hz	-0.184	-0.075	-0.038	-0.108	-0.073	-0.295	-0.240	-0.126
Hearing loss left 2048 Hz	-0.249	-0.229	-0.101	-0.197	-0.173	-0.194	-0.154	-0.125
Hearing loss left 4096 Hz	-0.210	-0.045	-0.140	-0.116	-0.006	-0.230	**-0.332***	-0.106
Start rate	-0.014	-0.053	-0.032	0.037	-0.220	-0.061	-0.097	0.072
Test motivation	-0.103	-0.163	-0.152	-0.103	-0.200	-0.102	-0.267	-0.032
Psychomotor endurance	0.000	0.019	-0.099	0.009	-0.052	-0.065	-0.253	-0.040
Viseomotor (time)	0.099	0.152	-0.096	0.148	0.154	-0.003	-0.083	0.009
Viseomotor (mistakes)	0.196	0.096	**0.346***	0.165	0.166	0.226	0.198	0.244
Optical reaction time	0.108	0.091	0.141	0.184	0.023	0.088	0.112	0.047
Acoustical reaction time	0.251	0.230	0.287	0.225	0.202	**0.404***	0.291	0.093

Pursuing reaction time	0.076	0.265	0.102	0.192	0.124	0.001	0.081	0.021
Verbal reaction time	0.268	0.240	0.299	0.205	0.223	0.169	0.126	0.262
Cognitive reaction time	0.091	0.133	0.203	0.134	0.104	-0.018	0.131	0.002
Cognitive switching	0.059	0.000	0.199	0.099	-0.017	-0.049	-0.020	0.095
Concentration (time)	**0.338***	0.199	0.306	0.238	0.268	0.285	0.186	**0.363***
Concentration (mistakes)	-0.163	0.044	-0.128	-0.036	-0.056	-0.219	-0.100	-0.128
Strategic thinking	0.252	0.189	**0.360***	0.253	0.131	0.304	0.264	0.267
Memory performance	-0.226	0.001	-0.090	-0.042	-0.096	-0.263	-0.050	-0.202
Orientation capability	-0.007	0.123	-0.040	0.109	0.037	-0.113	-0.154	0.057
Change over capability	0.308	0.169	**0.375***	0.223	0.144	**0.390***	**0.350***	0.276
Physical wellbeing	0.121	0.058	0.262	0.211	0.052	0.134	0.265	0.087
Emotional wellbeing	**0.381***	**0.416****	0.308	**0.476****	**0.427****	0.241	0.256	0.291
Overall wellbeing	0.199	0.146	**0.322***	0.286	0.142	0.168	0.286	0.157
Sense of coherence	**-0.517****	**-0.583****	-0.278	**-0.541****	**-0.629****	**-0.407***	-0.197	-0.289
Stress exposition	-0.173	-0.203	-0.195	-0.118	-0.233	0.035	-0.205	-0.138
Social dominance	**-0.452****	**-0.468****	-0.156	**-0.422****	**-0.531****	**-0.470****	-0.023	-0.213
Social power	0.010	0.022	-0.143	0.052	0.008	-0.043	-0.127	0.009
Stress predisposition	-0.248	-0.120	-0.010	-0.187	-0.144	-0.186	-0.026	-0.075
Social activity / duties	-0.038	0.044	-0.086	0.019	0.023	0.086	-0.174	-0.095
Social activity / leisure	0.026	0.054	0.015	0.089	0.123	0.011	-0.066	0.003

* = $p < 0.05$ / ** = $p < 0.01$
